# The multifaceted role of hair as a biospecimen: recent advances in precision medicine and forensic science

**DOI:** 10.1038/s12276-025-01548-4

**Published:** 2025-10-27

**Authors:** Sunil S. Adav, Kee Woei Ng

**Affiliations:** 1 School of Materials Science and Engineering, Nanyang Technological University, Singapore, Singapore; 2 Nanyang Environment and Water Research Institute, Nanyang Technological University, Singapore, Singapore

**Keywords:** Diagnostic markers, Breast cancer

## Abstract

Hair specimens are vital in precision medicine, forensics and environmental monitoring owing to their ability to retain biochemical data over time. Their noninvasive collection and long-term storage suitability make them ideal for diagnostics and investigations, offering historical insights into health and exposure records. In medicine, hair analysis provides a long-term biochemical profile, aiding in monitoring health conditions, nutritional deficiencies, toxin exposure and treatment efficacy. Advances in mass spectrometry, chromatography and spectroscopy have expanded their applications to cancer diagnostics, tuberculosis, HIV, neurological disorders and mental health assessments. In forensic science, the resistance of hair to decomposition and its ability to absorb substances help identify individuals, detect drug use and reconstruct crime scenes. Omics techniques such as genomics, proteomics and metabolomics enhance forensic accuracy by enabling precise substance detection and timeline reconstruction. Despite its potential, challenges such as hair growth variability, contamination and lack of standardized techniques limit the current impact of hair analysis. Addressing these issues could advance its role in diagnostics and forensic investigations. This review explores recent advancements and applications of hair analysis in precision medicine, infectious diseases, mental health, stress assessment and forensic science.

## Hair analysis revolutionizes medicine and forensic investigations

Hair is a simple yet valuable sample used in many fields such as medicine, cosmetics and forensics. It is easy to collect and store, making it useful for studying diseases and environmental exposures. This study aims to address how hair can be better used in precision medicine and diagnostics. Researchers have found that hair can help detect diseases such as cancer and monitor drug levels in patients. They use advanced techniques including mass spectrometry (a method to measure molecules) to analyze hair samples. This helps in understanding how drugs are absorbed and how diseases progress over time. Hair analysis can also reveal exposure to toxins and stress levels by measuring substances stored in the hair over months. The study concludes that hair is a promising tool for personalized healthcare, offering insights into long-term health conditions.

This summary was initially drafted using artificial intelligence, then revised and fact-checked by the author.

## Introduction

The hair shaft, despite its simplicity, is a valuable biospecimen in biological research, precision medicine and cosmetic science. Its versatility, noninvasiveness, easy collection, noninfectious nature and long-term storage suitability make it essential in forensic science, biomedical research, environmental studies, cosmetics and archaeology. Biospecimens such as tissue, blood, urine, skin and hair drive advancements in medicine, diagnostics and drug development. To ensure reliable results, the proper collection, processing and storage of specimens is crucial to prevent DNA, RNA and protein degradation while minimizing contamination. Unlike blood or tissue, which require complex biobanking, hair is easier to store. Advances in technology now prioritize ‘fit-for-purpose’ over ‘high-quality’ specimens^1^.

Hair is a valuable specimen for studying cosmetics, predicting age through DNA methylation and identifying biomarkers for cardiovascular health, aging and cancer^2,3^. It reveals metabolic changes linked to conditions such as obesity, Alzheimer’s disease, Parkinson’s disease, neurodegeneration, infant cognition and pregnancy complications^4^. In precision medicine, hair analysis supports drug-level monitoring, treatment adherence and metabolic assessment, especially in cancer care. The key applications include (1) genetic testing in oncology and neurology; (2) monitoring toxins, pollutants and heavy metals; (3) drug and metabolite tracking in chronic disease management; and (4) biomarker-based diagnostics for metabolic, neurological and stress-related disorders^5^. In forensic science, it is the most common biological specimen, aiding in determining sex, ethnicity, age, suspect identification, event reconstruction and drug-related crimes^6^.

**Fig. 1:**
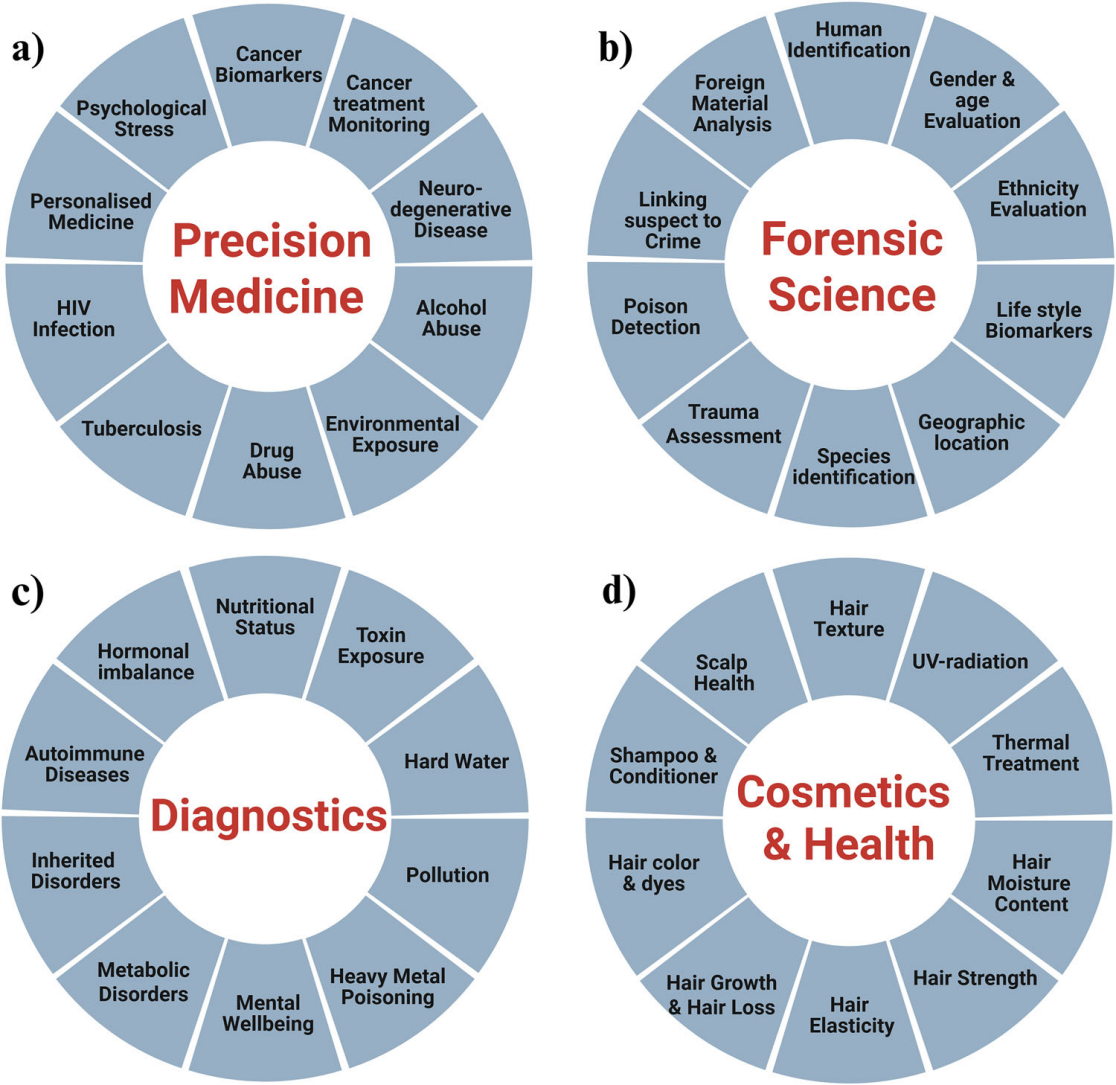
Diverse applications of hair biospecimens. Hair analysis provides valuable insights across multiple fields, including (**a**) precision medicine for individualized treatment, (**b**) forensic science for identity and evidence analysis, (**c**) diagnostics for disease detection and monitoring, and (**d**) cosmetics and health for assessing exposure and well-being.

Hair samples can capture various exogenous and endogenous exposures, including inorganic elements, drugs, tobacco and alcohol markers, pesticides, micro-organic contaminants and other toxic pollutants^7^. As a keratinized biological matrix composed mainly of keratin (65–95%), hair stores ingested substances, enabling retrospective drug analysis over months^8^. Consequently, hair serves as a valuable biospecimen for biomarker discovery, drug monitoring, toxicology, forensics, nutrition, endocrinology, psychiatry, neurology and environmental health. Figure 1 highlights its key applications across disciplines.

Recent technological advancements, notably in mass spectrometry (MS) and immunohistomorphometry, offer enhanced sensitivity and accuracy compared with traditional hair testing methods^2,9^. Building on traditional applications, the emerging roles of hair analysis (Table 1) underscore its expanding utility and importance as a retrospective biomonitoring tool, prompting a comprehensive review of recent advancements in the field. MS is highly utilized in various fields, including disease biomarker discovery, biotechnology, environmental monitoring, food processing, forensics, pharmaceuticals, agrochemicals and the cosmetics industry^10–13^. It is also adopted in hair analysis for analyzing biochemical, organic and inorganic compounds. Reports have highlighted advancements in nondestructive hair analysis through techniques such as Raman spectroscopy and infrared spectroscopy assays^14^, scanning electron microscopy^15,16^ and transmission electron microscopy. Artificial intelligence and machine learning are among the most exciting new developments in scalp and hair testing^17,18^.

**Table 1:** Traditional versus emerging roles of hair analysis.

Category	Traditional roles	Emerging roles
Primary purpose	–Forensic identification –Drug screening	–Clinical diagnostics and disease monitoring –Evaluation of treatment efficacy and adherence –Assessment of environmental and toxic exposures –Forensic and medico-legal analysis –Nutritional and metabolic health evaluation –Detection of illicit substances and metabolites –Specialized use in pediatric and psychiatric care
Analytical methods	–Light microscopy –Immunoassays –Basic chromatography –DNA profiling	–LC–MS/MS and HRMS for both targeted and untargeted compound analysis, including expanded panels for doping agents, opioids, antidepressants, novel psychoactive substances, steroids and low-dose medications –Matrix-assisted laser desorption/ionization−imaging MS and laser ablation inductively coupled plasma MS (LA−ICP−MS) for spatially resolved molecular and elemental profiling, enabling chemical ‘diaries’ of substance use, heavy metal exposure assessment and forensic geolocation –Isotope Ratio Mass Spectrometry (IRMS) and Surface Enhanced Raman Spectroscopy (SERS) for isotopic and vibrational molecular analysis in dietary studies, geographic origin tracing, doping control and in situ drug detection –Capillary Electrophoresis (CE)−MS for high-efficiency separation of charged biomolecules –Omics approaches, metabolomics, proteomics and genomics, for systems-level insights, biomarker discovery, and post-translational modification analysis –Ambient ionization techniques [for example, DART(Direct Analysis in Real-Time)–MS, Desorption Electrospray Ionization (DESI)–MS)] for rapid, in situ screening in forensic applications and security settings –Artificial intelligence and chemometric algorithms for high-dimensional data integration, interpretation and pattern recognition –Robotic hair sectioning and microsegmentation to enable high-resolution temporal and spatial profiling of substance use
Biological focus	−Morphological features (for example, color, thickness, curvature, texture and pattern) −DNA sequencing (if follicle present)	−Gene expression profiling −Protein quantification for assessing physiological states, and identifying biomarkers −Post-translational modification analysis −Metabolomic profiling to identify small-molecule biomarkers −Stable isotope ratio analysis for insights into diet, metabolic flux and environmental exposure
Forensic applications	−Personal identification −Drug abuse history	−Individual identification via GVPs and protein abundance profiling −Comprehensive toxicological screening for drugs, toxins and metabolites −Source attribution of chemical or environmental exposures to reconstruct exposure history −Behavioral and lifestyle reconstruction, substance use and physiological stress indicators −Stable isotope analysis to infer geographic mobility, diet and environmental context
Clinical applications	−Limited to drug monitoring −Rarely used due to reliance on blood/urine	−Biomarker discovery for early disease detection and risk stratification −Diagnostic support for cancer, psychiatric and neurodegenerative disorders −Therapeutic monitoring across conditions such as mental health disorders, cancer, TB, HIV, diabetes and thyroid dysfunction −Chronic stress assessment through long-term physiological marker integration −Hormonal profiling, including cortisol and other endocrine indicators −Therapeutic drug monitoring and evaluation of treatment adherence −Nutritional and metabolic status evaluation over extended periods −Illicit drug and metabolite detection for substance use assessment −Minimally invasive, longitudinal sampling for specialized pediatric applications
Toxicological applications	−Detection of drug/metabolite presence	−Quantitative longitudinal analysis for retrospective monitoring of substance exposure −Reconstruction of cumulative exposure to drugs, toxins, and environmental chemicals −Detection and profiling of NPSs −Pharmacokinetic and metabolism studies using time-resolved hair segment analysis −Assessment of prenatal exposure, including in utero transfer of drugs and toxins
Environmental applications	−Minimal or indirect	−Environmental pollutant assessment, including persistent organic pollutants and industrial chemicals −Heavy metal detection and quantification, such as lead, mercury and arsenic −Xenobiotic exposure analysis using stable isotope ratios and metabolite profiling −Elemental analysis for monitoring trace elements and environmental contaminants

Personalized hair testing is evolving, integrating genetics and medical history for more precise results in healthcare. This review provides a comprehensive overview of the emerging role of hair as a biospecimen in precision diagnostics. We highlight key scientific and clinical advancements driven by omics technologies, molecular diagnostics and artificial intelligence-enabled tools that are transforming hair analysis into a robust platform for biomarker discovery, treatment monitoring and disease stratification. Applications span oncology, infectious diseases, mental health, stress research and forensic science. By synthesizing developments across disciplines, this review underscores the growing utility of hair in precision diagnostics and personalized healthcare.

## The use of hair samples in precision medicine

Hair analysis dates back to the nineteenth century. In 1858, Hoppe^19^ discovered arsenic in the hair of a human corpse exhumed after 11 years. Nearly a decade later, amphetamine was detected in guinea pig fur by Goldblum et al.^20^. In the late 1970s, Baumgartner et al.^21^ developed the first radioimmunoassay kit for determining opiate abuse histories, marking the beginning of modern hair-based drug testing.

**Fig. 2:**
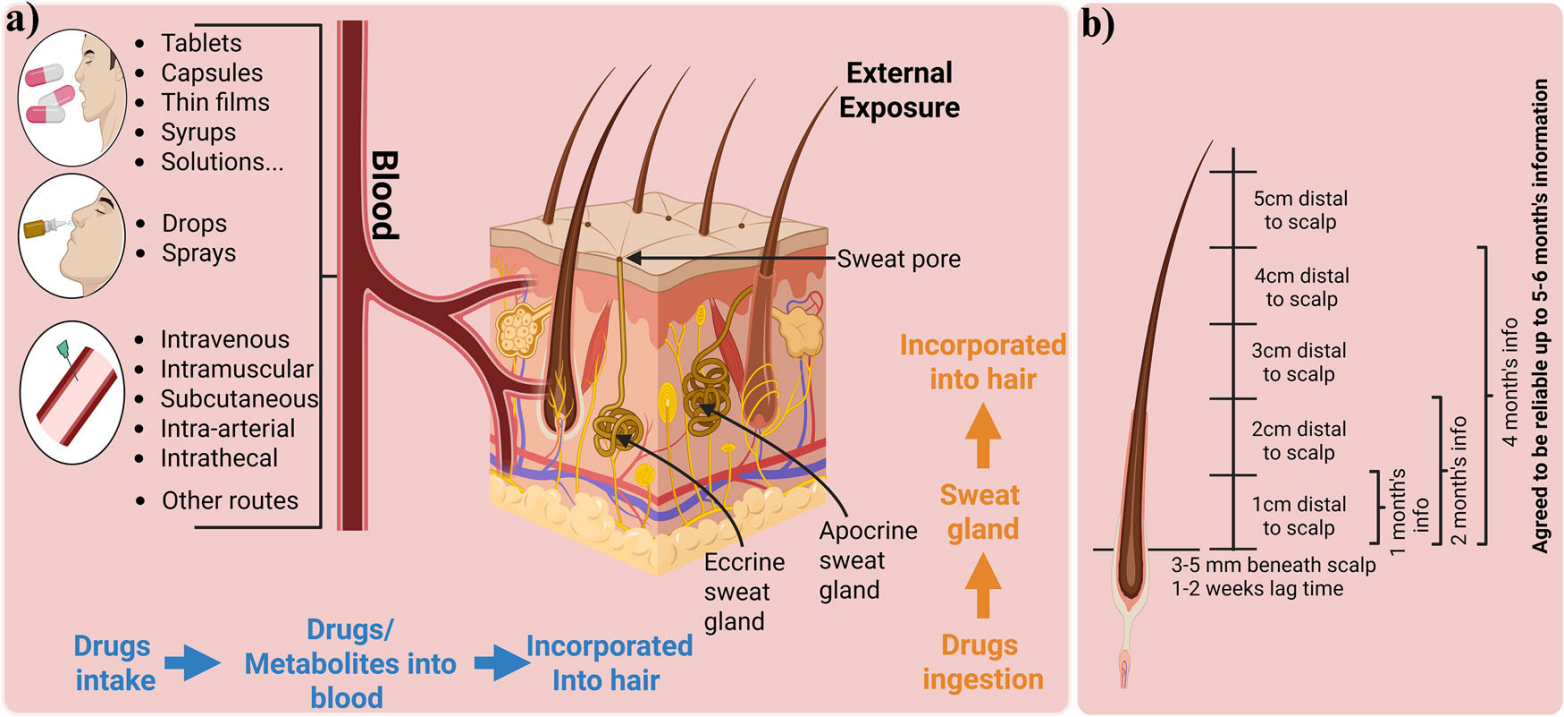
Routes and mechanism of drug and metabolite incorporation into hair. **a**, Routes of drugs incorporation into hair from blood, sebum, sweat and environmental exposure. **b**, Proposed mechanism for incorporation of drugs, metabolites or cortisol.

The precise mechanisms of drug incorporation into hair are not fully understood and require further research. However, recent experimental data support a complex multicompartment model for drug incorporation into hair (Fig. 2a). Drugs enter through (1) blood circulation during hair formation, (2) sweat and sebum after formation and (3) the external environment after the hair emerges from the skin. Additionally, substances can transfer from surrounding body compartments. The incorporation depends on the drug concentration in the blood, which reflects the ingested dose. Each scalp hair follows a growth cycle: anagen (active growth), catagen (regression) and telogen (resting) phase, lasting several years, a few weeks and several months, respectively^22^. Scalp hair grows at a rate of 0.6−1.4 cm per month, averaging 1 cm per month. As hair grows, substances are incorporated into the hair matrix, creating a chronological record of drug intake (Fig. 2b). Therefore, hair segmentation analysis is a valuable technique for evaluating a person’s drug administration history^23^.

The methodology for literature selection employed a structured narrative approach. Relevant peer-reviewed articles, reports and reviews were identified through database searches (PubMed, Scopus and Google Scholar) and manual screening of key journals and reference lists. Inclusion criteria prioritized studies aligned with this review’s objectives, methodological rigor and publication within the past 10 years. Non-peer-reviewed sources, studies lacking methodological detail and literature falling outside the thematic scope were excluded.

### Cancer management

**Fig. 3:**
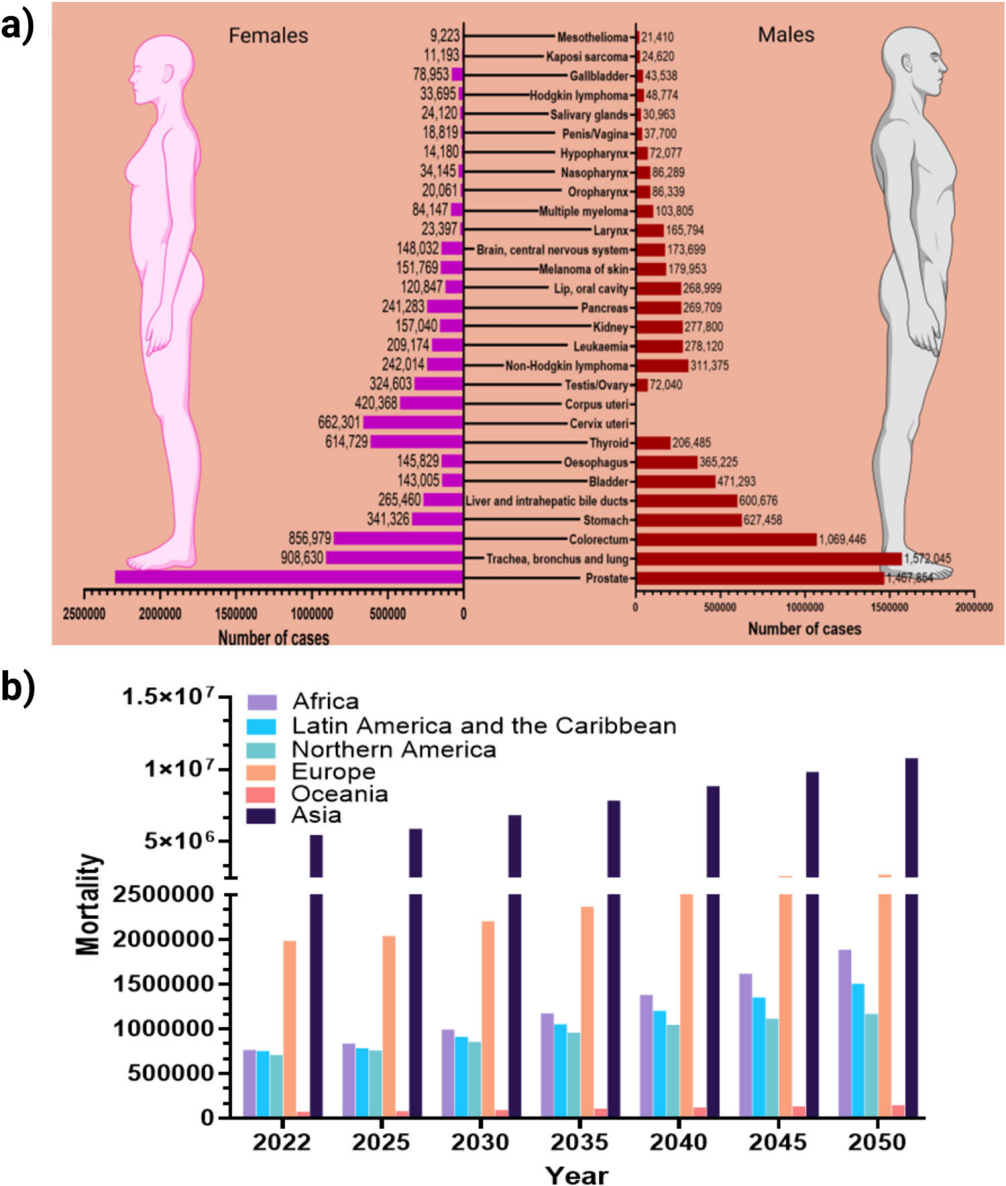
Cancer cases in both females and males in year 2022. **a**, New cancer cases in 2022. **b**, Mortality in 2022 and projection until 2050. Data sourced from WHO^25^.

Cancer is a major global public health problem and is the second leading cause of death^24^. Some cancer types with new cases in the year 2022 are shown in Fig. 3a, while the mortality projection until 2050 is in Fig. 3b (data source, WHO−IARC)^25^. The International Agency for Research on Cancer (IARC) projects that by 2040, there will be 30.2 million new cancer cases worldwide, with over 16 million expected deaths^25^. The diagnostics are crucial for accurate and early detection, personalized treatment planning and ongoing monitoring, while effective treatment is key to improving survival rates and quality of life for cancer patients. The role of hair biospecimens in diagnostics and treatment monitoring is discussed below.

#### Cancer diagnostics

The gold standard for cancer diagnosis often requires a combination of biopsy with histopathological examination, laboratory tests and sometimes genomic profiling. These methods, while essential, are invasive and time consuming. Alternative imaging methods such as X-rays, computed tomography scans, magnetic resonance imagings and positron emission tomography scans suffer from low sensitivity and high false-positive rates. While blood and tissue biopsies are commonly used, their limitations in sampling frequency and storage highlight hair analysis as a valuable complementary approach.

James^26^ studied hair using X-ray diffraction (XRD) studies with synchrotron radiation and found that hair from patients with breast cancer has a different intermolecular structure from that of healthy subjects. However, the other researchers failed to reproduce data using this technique^27,28^. Since 1999, over 500 hair samples have been analyzed by the James group in double-blinded breast cancer studies with no false negatives being detected. The analysis of pubic hair yielded similar results, leading to the proposal of using pubic hair as an alternative to avoid interference caused by cosmetic treatments on scalp hair. In 2005, James et al.^29^ provided additional evidence using an animal model of breast cancer. They analyzed whiskers from nude mice, removed before and 8 weeks after subcutaneous implantation of a human breast adenocarcinoma cell line. Postimplantation whiskers displayed a ring in the XRD pattern, similar to that in human breast cancer patients. This ring appeared within 2 weeks of implantation, before any visible tumor formed, indicating that changes in hair could serve as an early cancer marker. Evidence for this finding has been supported by studies on the molecular nature of the structural alteration^30,31^ and clinical testing^28^. Synchrotron XRD offers enhanced sensitivity and accuracy over conventional XRD due to its high intensity, collimated and tunable beams, enabling trace-phase detection, high-resolution structural analysis and element-specific measurements, particularly in small-volume or complex samples. Han and colleagues^32^ observed medulla loss and cortical membrane enhancement in the hair strands of patients with breast cancer that revealed structural variations, suggesting the potential application of synchrotron X-ray imaging in breast cancer screening.

Fourier transform infrared (FTIR) spectroscopy is a valuable tool for analyzing hair in medical research, especially for cancer detection. By assigning wavenumbers to biomolecules and their interactions (Supplementary Table 1), FTIR generates molecular fingerprints that reveal systemic physiological changes, including those linked to cancer^33^. FTIR attenuated total reflection analysis of scalp and pubic hair from patients with breast cancer showed increased β-sheet/disorder protein structures and elevated C–H lipid content^34^, indicating disease-related alterations in hair composition. Consistently, Lyman and Fay^35^ observed enhanced peak ratios in the 1,446−1,456 cm^-1^ C−H bending region of scalp hair from these patients. KRT81, a type II hair keratin normally expressed in the hair cortex, has been detected in the SKBR3 breast cancer cell line^36^ and metastatic lymph nodes of breast carcinomas^37^, but not in normal breast epithelial cells, indicating its potential as a breast cancer biomarker.

Wu et al.^38^ explored the use of synchrotron radiation infrared microspectroscopy on hair specimens to differentiate patients with esophageal cancer from healthy individuals. Analyzing spectral peaks and performing discriminant analysis on the hair medulla of 39 patients and 32 healthy subjects, they found that the hair medulla could effectively distinguish between the two groups. They achieved a sensitivity of 89.74%, specificity of 87.50%, positive predictive value of 89.74% and accuracy of 88.73%. Similar studies on colorectal cancer^39^ and lung cancer^40^ revealed significant changes in hair protein, lipid and nucleic acid profiles, suggesting these alterations reflect underlying cancer pathology. In addition to these cancers, the pulmonary fibrosis diagnosis and progression could be accurately evaluated from the hair metabolome^41^. These authors proposed ornithine, 4-(methylnitrosamino)-1–3-pyridyl-*N*-oxide-1-butanol, desthiobiotin and proline as diagnostic metabolites, and azelaic acid, indoleacetyl glutamic acid and cytidine as progression markers. Cervical cancer, the fourth most common cancer, affected an estimated 662,301 women and caused 348,874 deaths globally in 2022^25^. Ran et al.^3^ examined hair metabolomics of patients with cervical cancer by comparing metabolite profiles in hair, cervical tissue, plasma and urine. Hair and cervical tissue showed the clearest distinction between cancer and control groups. Among 181 metabolites in hair, eight—including amino acids (alanine, aspartic acid, glutamic acid, glycine and phenylalanine), norvaline and organic acids—were elevated in patients with cancer. Pathway analysis linked these metabolites to tumor-associated metabolic processes.

Trace elements, present in low concentrations in the human body, are essential for physiological functions such as antioxidant defense, immune response, redox signaling, wound healing and gene regulation^42^. Key trace elements such as iron, zinc, iodine, fluoride, copper, potassium and calcium play crucial roles in health (Sugimoto et al.^43^) and are tightly regulated by homeostasis^43^. Deviations from normal levels or range or deficiencies can lead to nonspecific symptoms, organ dysfunction, congenital malformations and disease^44^. For example, low zinc (Zn) levels have been linked to various cancers including esophageal, breast, liver, lung, gynecological, colon, oral, prostate and pancreaticobiliary^45,46^ and many more as reviewed by Sugimoto et al.^43^. Hair element levels, which mirror blood concentrations, remain stable in healthy individuals. In patients with oncological mammary pathology, scalp hair analysis has shown a significant decrease in the concentrations of selenium (Se) and Zn, along with an increase in chromium (Cr) levels^47^. Nejad and colleagues^48^ reviewed 52 studies with 163,909 participants and found that lower Zn levels in scalp hair were linked to prostate cancer.

In testing the feasibility of applying trace element analysis of hair to accurately distinguish patients with prostate cancer from healthy individuals, Tan and Chen^49^ demonstrated that trace element analysis of scalp hair, including nine elements (Zn, Cr, Mg, Ca, Al, P, Cd, Fe and Mo), achieved 98.2% accuracy. Qayyum and Shah^50^ found elevated levels of Fe, Mn, Ni, Cr and Pb in the scalp hair of patients with prostate cancer. Multiple studies from countries such as Turkey^51^, India^52^, Italy^53^ and Poland^54^ have explored this relationship between scalp hair trace element levels in patients with cancer. In breast cancer, Gholizadeh et al.^55^ used proton-induced X-ray emission to measure scalp hair element levels in healthy women, those with benign breast disease and patients with breast cancer. They found that while S, Cl, K, Ca, Fe and Cu concentrations were similar across healthy, benign and patients with cancer, lower Zn levels were seen in hyperplasia and patients with breast cancer.

The level of heavy metals and trace elements such as Li, Ag, Pd, Ti, Co, Ni, As, Sn and so on, in hair has been proposed as biomarkers of lung cancer^51^. Wozniak et al.^56^ reported significant differences in essential (Ca, Mg, Zn, Cu, Fe and Mn) and toxic metal (Pb, Cd, Co and Cr) concentrations in the hair of patients with head and neck cancer compared with healthy controls, indicating disrupted metal homeostasis. Patients with thyroid cancer showed elevated levels of Mn, Co, Cr, K, Fe, Mg, Pb, Na and Ni, but lower Zn levels^57^ in scalp hair. Patients with stomach cancer patients had higher Cr levels, while Fe, Mn, and Cd were lower in scalp hair^58^. Patients with gastrointestinal stromal tumors had elevated Pb levels, while stage III patients showed higher Fe and Pb, and stage IV had higher Cr in scalp hair. Similar findings have been reported in laryngeal^59^, prostate^60^ and other cancers^56^. These studies suggest that hair element analysis holds promise as a diagnostic tool for cancer detection.

Emerging research underscores the potential of hair metabolomics and epigenetic markers for early cancer detection, especially in breast, colorectal and prostate cancers. However, challenges include limited sensitivity for low-abundance targets, susceptibility to external contamination and a lack of standardized protocols. With ongoing methodological advancements, hair biospecimens represent a scalable, patient-centric platform for integrative oncology, linking pharmacologic, environmental and molecular data to better understand cancer risk, progression and therapeutic response. Although still in development and requiring further validation, hair analysis holds promise as a valuable tool in personalized medicine for cancer diagnosis and monitoring.

#### Tracking cancer treatment efficacy

Cancer treatment involves a range of drugs targeting cancer cells, the tumor environment and patient-specific factors. Key therapies include chemotherapy, hormone therapy, targeted therapy, immunotherapy, radiopharmaceuticals, bisphosphonates, PARP inhibitors, histone deacetylase inhibitors and angiogenesis inhibitors. In addition to FDA-approved drugs, new treatments and combinations are being developed to address rising cancer rates and improve survival and quality of life. However, managing severe side effects, such as nephrotoxicity, neurotoxicity, ototoxicity and myelosuppression, remains a challenge. Nonspecific chemotherapies can damage healthy tissues and improper dosing may compromise patient survival. As cancer prevalence rises, the demand for effective treatments will continue to grow. Hence, monitoring treatment effectiveness is crucial for optimizing dosing, minimizing side effects and ensuring treatment adherence. Hair, a noninvasive specimen, provides valuable insights into drug efficacy, resistance, treatment adjustments and toxicity. Its slow growth offers a chronological record of treatment exposure and drug metabolism, making it promising for personalized medicine. Although still emerging, using hair for cancer monitoring could greatly enhance patient care and lead to more precise treatment management with further research.

The platinum-based anticancer drugs such as cisplatin (*cis*-diamminedichloroplatinum(II)), carboplatin, oxaliplatin and satraplatin, with manifest therapeutic effects and well-defined mechanisms of action^61,62^ are frequently employed in adult oncologic treatment protocols in treating diverse cancers, such as breast, ovarian, colorectal, testicular, head and neck cancers^63^. Cisplatin, carboplatin and oxaliplatin have similar pharmacodynamics but distinct antineoplastic activities. Therefore, they are used in different treatment protocols for tumors in both pediatric and adult populations. Using laser ablation inductively coupled plasma MS (LA−ICP−MS), Pozebon et al.^64^ monitored the platinum levels in the hair of a 58-year-old patient with ovarian cancer treated with four cycles (days 0, 25, 46 and 73) of cisplatin/cyclophosphamide (75 mg/750 mg). Hair samples collected on day 120 revealed four platinum peaks corresponding to the treatment doses at 3-week intervals. The Pt signal maxima occurred at hair length intervals of 6.8 mm between the first and second doses, 6.2 mm between the second and third doses, and 7.9 mm between the third and fourth doses. This analysis provides a clear temporal record of cisplatin administration.

FTIR analysis of hair samples from 82 patients with lung cancer showed significantly lower protein and lipid levels^40^. These authors also monitored a 76-year-old male with extensive-stage squamous cell carcinoma undergoing chemotherapy (paclitaxel liposome 210 mg + carboplatin 450 mg) and immunotherapy (camrelizumab 200 mg). Over 4 months of chemotherapy and immunotherapy, computed tomography scans, blood tests and hair analysis tracked treatment. The tumor size decreased and serum total protein levels improved with each chemotherapy session, indicating health improvement. Hair FTIR peaks at 2,961 cm^−^¹, 2,931 cm^−^¹, 1,452 cm^−^¹, 1,397 cm^−^¹ and 1,238 cm^−^¹ increased, correlating with his treatment response and health recovery. They monitored another patient on treatment (paclitaxel liposome 180 mg + carboplatin 600 mg) and immunotherapy (sintilimab 200 mg), which a showed similar trend, confirming hair analysis as a valuable tool for monitoring lung cancer chemotherapy.

Imatinib is the first-line treatment for chronic myeloid leukemia, with a target concentration of 1,000 ng/ml for effective therapy. Noncompliant patients often take medication more consistently before physician visits, leading to inaccurate blood drug levels. Factors such as high costs, complex regimens, side effects, mental health issues, forgetfulness, intentional noncompliance, limited education and lack of support contribute to nonadherence. Random dried blood spots (DBSs) or hair samples provide a more accurate assessment of adherence. Hair drug concentrations correlate with therapeutic response^65^. Capron et al.^66^ used liquid chromatography−tandem MS (LC−MS/MS) to measure imatinib levels in hair samples from 102 patients with chronic myeloid leukemia treated for at least 4 months. Patients with therapeutic failure exhibited lower hair IM concentrations, indicating hair analysis as a potential tool for monitoring treatment adherence.

Tyrosine kinase inhibitors (TKIs) are first-line small-molecule drugs in cancer therapy, inhibiting abnormal cell growth by targeting tyrosine kinase signaling. While plasma is the standard for TKI monitoring, a robust LC‒MS/MS method has quantified erlotinib in scalp hair^67^. Similarly, tamoxifen, a key drug for hormone-sensitive breast cancer, has been detected in hair samples^68^. Given the long-term use of TKIs, adherence is vital. Segmental hair analysis provides a chronological profile of drug concentrations, enabling detection of the timing and duration of missed doses while minimizing the need for repeated blood sampling.

Anticancer drugs, while therapeutic, pose occupational risks for hospital workers, with traces found in urine and blood. Exposure has been linked to adverse reproductive outcomes, including spontaneous abortion, premature delivery and low birth weight^69^. Hori et al.^70^ used hair samples and ICP−MS to monitor platinum-based drug exposure in hospital staff. Additionally, measuring scalp hair cortisol levels can track the clinical course and treatment response in patients with (cyclic) Cushing syndrome^71^.

In summary, hair is a stable biospecimen that can be collected minimally invasively, with growing applications in oncology for diagnostic and therapeutic monitoring. Unlike blood or urine, hair captures long-term biochemical exposures through continuous growth, enabling retrospective, time-resolved analysis of chemotherapeutics, endocrine disruptors, heavy metals and carcinogens. This longitudinal insight supports the evaluation of treatment adherence, dose optimization and cumulative risk, particularly in pediatric, occupational and resource-limited settings. However, the use of hair as a biospecimen presents several analytical, technical, environmental and other limitations, as outlined in ‘Challenges and limitations’ section.

### Insights into neurological disorders

Aging is the leading risk factor for neurodegenerative diseases such as Alzheimer’s disease (AD), Parkinson’s disease, Huntington’s disease, amyotrophic lateral sclerosis, multiple sclerosis and Creutzfeldt−Jakob disease^10,72^. AD is marked by tau and amyloid plaque buildup, Parkinson’s disease by α-synuclein accumulation causing dopamine cell loss and Huntington’s disease by Huntingtin (Htt) aggregates. Biomarker discovery for AD typically relies on plasma, saliva, urine, cerebrospinal fluid and postmortem brain tissue^73–75^, although these are influenced by circadian rhythms, daily activities and even sleep patterns^76^. Hair, however, is emerging as a stable alternative biospecimen. Tan et al.^77^ used LC‒MS/MS to analyze metabolic changes in rat hair after β-amyloid (Aβ1-42) exposure. After 35 days, rats showed cognitive deficits, with 40 altered metabolites. Twenty were linked to three disrupted pathways: (1) phenylalanine metabolism and related biosynthesis, (2) arachidonic acid metabolism and (3) unsaturated fatty acid biosynthesis. Large-scale studies could improve the diagnostic potential of these AD biomarkers. The 5xFAD mouse model, carrying three amyloid precursor proteins and two presenilin 1 mutations, is widely used to study Aβ accumulation^78^. Chang et al.^79^ applied high-resolution MS (HRMS)-based metabolomics on hair samples from 6-month-old 5xFAD and wild-type mice, identifying 45 differential metabolites and three disrupted pathways: arachidonic acid, sphingolipid and alanine, aspartate, and glutamate metabolism.

Evidence links AD to abnormal lipid metabolism, particularly cholesterol and its esters^80^, which may drive amyloid plaque formation. Cholesterol regulates both Aβ production and clearance. Elevated cholesterol levels increase Aβ accumulation, while cholesterol-lowering drugs reduce it^81^. Son et al.^82^ developed a GC−MS-based hair profiling technique to analyze 16 endogenous sterols in control individuals and patients with mild cognitive impairment and AD. Their findings showed that cognitive impairment correlated with an increased metabolic rate, notably elevated 7β-hydroxycholesterol levels. Su et al.^83^ analyzed hair metabolites in patients with AD and matched control individuals using HRMS‒based untargeted metabolomics, identifying 25 discriminatory metabolites. Several, including acetyl-l-carnitine, propionylcarnitine, butyrylcarnitine, *O*-valeroyl-l-carnitine and LPC 18:1, showed reduced levels in patients with AD. A panel of five metabolites: 6-*O*-methylnorlaudanosoline, acetyl-l-carnitine, propionylcarnitine, butyrylcarnitine and *O*-valeroyl-l-carnitine, was proposed as potential early AD markers, aligning with strategies favoring biomarker panels over single markers^84^. The reduced levels of 6-*O-*methylnorlaudanosoline, a norlaudanosoline derivative that contributes to oxidative stress and synaptic dysfunction, in patients with AD may indicate increased neurodegeneration, supporting its potential as an early AD marker in hair^85^. Chang et al.^86^ further analyzed hair samples from patients with AD and control individuals using data-dependent acquisition, full scan with targeted MS/MS, and all ion fragmentation. These methods identified 15,732, 23,196 and 20,902 aligned features with 41, 407 and 366 discriminatory features, respectively. Further analysis highlighted five candidate biomarkers including *N*-dodecanoyl-*N*-methylglycine, 15-methyl-15R-PGF2α methyl ester, dihydrophaseic acid, valeryl-carnitine and 7α,12α-dihydroxy-3-oxo-4-cholenoic acid as potential AD biomarkers. Large-scale validation is needed to confirm their diagnostic utility.

Metals such as Cu, Zn and Fe are vital for enzyme function, neurotransmission and aging. However, excessive exposure causes oxidative stress, disrupting neurodegenerative genes through epigenetic mechanisms, contributing to late-onset neurodegenerative diseases^87^. Cicero et al.^88^ reviewed associations between metals and neurodegenerative disorders, including amyotrophic lateral sclerosis, AD and Parkinson’s disease. A Spanish study linked Cr exposure to increased mortality from motor neuron disease^89^. Arain et al.^90^ found significantly higher Al and Mn levels in scalp hair samples of patients with neurological disorder compared with control individuals. Another study on psychiatric disorders revealed increased Al and Mn levels in patients with psychiatric disorders^91^. AD is associated with dysregulated Cu, Zn, Fe and other elements such as Al, Mn, Pb and Cd, which co-localize with Aβ protein in plaques^92^. Metals play a key role in AD and plaque formation, prompting the proposal of chelation therapy to combat metal-induced neurodegeneration. Singh et al.^93^ reviewed recent literature on metal-based theranostic agents, including metal chelators, complexes and nanoparticles for AD therapy. A Cu, Fe and Mn imbalance triggers oxidative stress, promoting α-synuclein aggregation^94^. To test the link between hair and Parkinson’s disease, Jucevičiūtė et al.^95^ enrolled patients with Parkinson’s disease and age and gender-matched Parkinson’s disease-unaffected individuals, and found earlier onset of hair graying and dysregulated sebum production, while another study revealed altered Ca and Mg levels in hair samples from patients with Parkinson’s disease across disease stages^96^. In multiple sclerosis, a comparison of hair samples from patients with multiple sclerosis and control individuals revealed significant differences in Al, Rb and U levels, with higher Ag, Cr, Fe, Ni and Sr in female patients with multiple sclerosis^97^. Hair follicles, sharing an ectodermal origin with the brain, serve as potential diagnostic tools for brain diseases. Transcriptomic profiling of hair follicles has been explored for identifying biomarkers in autism, chronic psychosis and methamphetamine use disorder^98^. Elevated levels of metals such as Pb and Zn in hair have been linked to increased risk of Parkinson’s disease^99^. Additionally, hair metabolomics has revealed AD-specific metabolic alterations and oxidative stress markers that may precede clinical symptoms^79,83^. These findings support the utility of hair as a complementary tool for population screening, epidemiological surveillance and longitudinal monitoring in clinical research. In summary, hair remains a valuable biospecimen in neurological disorders and diagnostics.

### Monitoring TB treatment

Tuberculosis (TB) is a preventable and curable disease, yet it remains the second leading cause of death from a single infectious agent globally, after COVID-19, with nearly 1.3 million deaths in 2022^100^. Over 10 million people contract TB annually. The standard 6-month treatment for drug-susceptible TB is complex and often leads to treatment defaults and resistance. Recently, the WHO introduced a new 4-month regimen, but it is not widely adopted. TB treatment is further complicated in HIV co-infected patients due to pill burden, drug toxicities and poor absorption, increasing mortality^101,102^. Subtherapeutic drug levels are common, especially in multidrug-resistant TB and HIV co-infection, contributing to resistance and poor outcomes. Recent guidelines from the American Thoracic Society and other health organizations recommend therapeutic drug monitoring to optimize dosing in both drug-susceptible and drug-resistant TB cases^103,104^.

Monitoring TB treatment adherence using blood plasma or serum drug levels is considered the gold standard, but daily sampling makes it impractical. Less invasive samples such as DBSs, urine, saliva and hair are becoming popular due to easier collection and storage. While urine, saliva and DBS provide drug concentrations at specific time points, they cannot capture cumulative exposure over the entire treatment. Hair samples, however, can offer a more accurate reflection of long-term drug exposure, even for short half-life drugs such as isoniazid and linezolid^105^. This method also provides insights into adherence patterns, which is crucial for prolonged anti-TB treatment.

Mave et al.^106^ analyzed isoniazid concentrations in the hair of children under 12 years with TB, using LC−MS/MS to assess adherence and exposure-response at 1, 2, 4 and 6 months. The detectable levels helped evaluate treatment adherence and exposure−response (pharmacokinetics/pharmacodynamics) relationships. Later, these authors^105^ measured TB drug concentrations in hair samples from children and adults in Pune, India, finding that higher isoniazid and acetyl-isoniazid levels were linked to better treatment outcomes and reduced TB treatment failure. Metcalfe et al.^107^ developed an LC‒MS/MS assay to measure multiple (isoniazid, pyrazinamide, ethambutol, levofloxacin, moxifloxacin, bedaquiline, clofazimine, linezolid, ethionamide and pretomanid) anti-TB drug concentrations in hair, aiming to predict outcomes for extensively drug-resistant TB. This assay was later validated for use with small hair samples^108^. LC−MS/MS offers high accuracy (typically within 5–10% of true values) and resolution for polar, thermally labile and biologically relevant compounds, with minimal sample preparation. GC−MS/MS provides excellent resolution for volatile, low-molecular-weight analytes but often requires derivatization to enhance volatility and stability. A recent review^109^ confirmed hair’s potential for multi-analyte drug monitoring but highlighted the need for large-scale validation studies.

Hair-based drug monitoring has demonstrated utility in clinical trials and real-world multidrug-resistant tuberculosis programs across South Africa, India and Vietnam. Studies by Metcalfe et al.^110^ and Rao et al.^109^ showed that hair concentrations of anti-TB drugs enabled individualized dosing, adherence monitoring and detection of subtherapeutic exposure, particularly in patients with inconsistent adherence. Similarly, initiatives in Uganda, Kenya and China used hair antiretroviral (ARV) levels to inform clinical decisions and monitor pre-exposure prophylaxis (PrEP) adherence. In pregnancy cohorts, hair sampling offered a low-risk method for assessing maternal–fetal drug exposure^111,112^.

### HIV treatment monitoring

Over 20 million people with HIV are on ARV therapy (ART), with many more expected to start soon. ART effectively lowers viral load to undetectable levels, preventing transmission and improving quality of life. HIV drugs are classified into several categories: (1) nucleoside reverse transcriptase inhibitors (NRTIs), (2) non-NRTIs, (3) protease inhibitors, (4) integrase strand transfer inhibitors, (5) fusion Inhibitors (enfuvirtide (T-20)), (6) CCR5 antagonists and (7) pharmacokinetic enhancers. HIV medications are available as combination pills, with proven effectiveness. Daily emtricitabine/tenofovir disoproxil fumarate (FTC/TDF) as PrEP reduces HIV-1 acquisition in men^113^. Both ART and PrEP require strict adherence for effectiveness.

Improving medication adherence is crucial for reducing HIV transmission and enhancing health outcomes. Long-term ART use can be challenging due to complex regimens and nonadherence, leading to virologic failure, drug resistance and side effects. Monitoring adherence is essential for those on ART, PrEP or other medications. Traditional methods (for example, self-reports or pill counts) are limited in accurately measuring adherence. Spinelli et al.^114^ proposed new approaches, including pharmacologic measures, electronic monitors and ingestible pills. Adherence factors vary by treatment, dosing, socioeconomic status and individual circumstances. Although there is no gold standard, testing drug levels in biological samples provides more accuracy. Short-term monitoring uses plasma, urine or saliva, while hair analysis offers long-term data^65^. Bernard and colleagues^115^ found that indinavir levels in hair correlated closely with the extent of HIV suppression. Liu et al.^65^ investigated the relationship between dose and tenofovir concentrations in hair among healthy, HIV-uninfected adults, finding a strong correlation between dosing frequency and hair tenofovir levels. This suggests that quantitative measuring drug levels in hair could improve adherence assessment and help establish exposure thresholds.

Virologic failure often indicates poor adherence to ART. Low ARV concentrations in hair suggest nonadherence, while high levels warrant viral resistance testing. Early measurement of hair ARV levels can identify patients at risk for treatment failure. Gandhi et al.^116,117^ demonstrated that hair ARV concentrations strongly predict virologic outcomes in a large clinical trial. According to literature^118,119^, hair ARV concentrations reflect long-term pharmacokinetic parameters influenced by biological factors and average adherence over time. Apornpong et al.^120^ analyzed ARV hair concentrations in participants on two NRTIs with boosted atazanavir or lopinavir at weeks 12, 24, 36 and 48 using LC−MS and found that protease inhibitor concentrations in hair correlated more strongly with virologic outcomes than self-reported adherence, highlighting hair as a tool for identifying those at risk of second-line failure. In a cohort of 271 HIV-infected women receiving nevirapine, Baxi et al.^121^ found that nevirapine hair levels strongly predicted virologic suppression, reinforcing hair as a valuable biospecimen. Zhang et al.^122^ reviewed 31 studies on 11 ARVs across four drug classes (NRTIs (3TC, TFV and FTC), non-NRTIs (NVP and EFV), protease inhibitors (IDV, ATV, LPV, RTV and DRV) and integrase strand transfer inhibitors (RAL)), finding strong associations between hair ARV concentrations and pharmacokinetic adherence measures, as well as pharmacodynamic responses (viral load and toxicity). This supports hair ARV levels as a reliable, objective marker of long-term adherence.

In HIV care, hair analysis has emerged as a robust method for assessing long-term adherence to ART, with concentrations of drugs such as tenofovir, emtricitabine, efavirenz and lopinavir showing strong correlations with virologic suppression and clinical outcomes^123^. Compared with self-reports or pill counts, hair levels provide more objective, cumulative adherence data. Hair-based metrics have been integrated into HIV cure-related trials and real-world implementation programs, offering scalable, noninvasive and ethically acceptable adherence monitoring^124^. By overcoming logistical and behavioral barriers to traditional methods, hair analysis may enhance precision in HIV treatment monitoring and support optimized, patient-centered care.

### Assessing mental health and stress levels

Chronic stress significantly impacts health and mental illness. Cortisol, a key stress hormone regulated by the hypothalamic−pituitary−adrenal (HPA) axis, maintains homeostasis by influencing blood pressure, metabolism and glucose levels^125^. While short-term stress aids balance, prolonged activation disrupts neurogenesis, synaptic plasticity and structural integrity, increasing neuropsychiatric risks^126^. Chronic stress damages tissues, desensitizes receptors and heightens risks for conditions such as diabetes, chronic pain, obesity, cardiovascular diseases and mental disorders^127,128^.

Traditionally, cortisol is measured in saliva, blood or urine, but yield inconsistent results^129^, whereas hair cortisol concentration (HCC) offers a long-term assessment, aiding studies on chronic conditions such as Cushing’s disease, depression and chronic pain^127^. Research suggests that HCC is unaffected by hair treatments but tends to increase with age and in males^130,131^. Elevated cortisol, especially in childhood, adolescence and late adulthood, correlates with depression and stress disorders^132^. Pandemics such as COVID-19 exacerbate stress, disrupting HPA function and increasing mental health issues, including post-traumatic stress disorder, depression and anxiety. Key stressors include quarantine, infection fears, financial strain and stigma^133^. A review of 24 studies confirmed the negative psychological impact of quarantine^133^. Studies show that 40% of healthcare workers during COVID-19 had altered HCC levels, linking elevated HCC to depressive symptoms^134^. HCC analysis has emerged as a valuable biomarker for chronic stress, linking it to depression, post-traumatic stress disorder and suicidality^135^. Integrating HCC profiling into clinical practice may enhance mental health diagnosis and treatment. Table 2 provides a summary of research that uses hair samples to examine hair cortisol levels, chronic stress and mental well-being. Studies link elevated HCC in pregnant women to increased stress, anxiety, depression and potential negative impacts on maternal−infant bonding and infant development^136^. Caparros-Gonzalez et al.^137^ found significant differences in HCC between the first and third trimesters, suggesting a predictive role for postpartum depression. A meta-analysis of 29 studies found stronger associations between maternal psychological distress and HCC during pregnancy than postpartum^136^. Additionally, maternal stress correlates with infant gut microbiota composition^138,139^. Further, HCC level has been used to assess stress in students, teachers, workplace environments, war exposure and post-traumatic stress^140^ and so on. It has also proven effective in evaluating chronic stress in war victims and refugees.

**Table 2:** Summary of research utilizing hair samples to investigate hair cortisol, chronic stress and mental well-being.

Research Topic	Method	Sample	Summary of research finding	Reference
Intensive aerobic exercise, endurance sports	−Cortisol levels in the first to third 3-cm-long hair segments closest to the scalp −Method includes hair segment washing with isopropanol, ball mill and steroid extraction using methanol, immunoassay (CLIA)	−Hair samples were from 304 amateur endurance athletes (long-distance runners, triathletes and cyclists) and 70 control individuals. Hair from the posterior vertex as close to the scalp	−The physical stress from intensive training and competitive races in endurance athletes was linked to increased cortisol levels	^189^
Chronic stress on cardiovascular risk (coronary heart disease, stroke, peripheral arterial disease, diabetes mellitus and other chronic, noncardiovascular diseases)	−Cortisol levels in the 3-cm-long hair segment. Method includes hair segment washing and steroid extraction using methanol, ELISA kit (DRG Instruments)	−Approximately 150 hair strands cut from the posterior vertex, as close to the scalp from 283 participants	−High hair cortisol levels with an increased cardiovascular risk −No associations between hair cortisol levels and noncardiovascular diseases	^190^
COVID-19 pandemic (sense of coherence, mental health)	−Cortisol levels in 3-cm hair. Method includes hair segment washing with isopropanol, ball mill and steroid extraction using methanol, radioimmunoassay (Orion Diagnostica)	−Hair samples were from 260 participants. Hair from the posterior vertex as close to the scalp	−Sense of coherence was significantly associated with anxiety mental health	^191^
COVID-19 pandemic (depressive symptoms), mental health	−Cortisol levels in the 3-cm-long hair segment. Method includes hair segment washing and steroid extraction using methanol, LC–MS/MS	−Hair samples from 1,025 adults. Hair from the posterior vertex as close to the scalp	−Cortisol was positively and significantly associated with elevated depressive symptoms during the COVID-19	^192^
COVID-19 pandemic (stress, and burnout, psychological distress, and any other mental health in a health workers)	−Cortisol levels in the 3-cm-long hair segment. Automated chemiluminescent method (Immulite 2000 autoanalyzer)	−Hair samples from 234 (68 men and 166 women) health workers from Hospital. Hair from the posterior vertex as close to the scalp	−Higher values in hair cortisol levels in the group with burnout. An association between perceived stress and hair cortisol levels was observed	^134^
Chronic stress (myocardial infarction)	−Cortisol levels in the 1-cm-long hair segment. Method includes hair segment washing, ball mill and steroid extraction using methanol	−Hair samples from myocardial infarction [(acute myocardial infarction (AMI), *n* = 174)] and 3156 control individuals	−Higher levels of HCC were strongly and statistically significantly associated with current AMI status.	^128^
Chronic stress and hair cortisol in children	−Extracting Cortisol from 1, 3 or 6 cm of hair, measuring HCC by LC−MS/MS	−Data from five countries with 1,455 participants.	−This review of nine studies found significant positive correlations between chronic stress, measured by stressful life events in the past 6 months and HCC	^141^
Chronic stress (angiographically confirmed coronary atherosclerosis)	−HCC was assessed from scalp hair by ELISA	−500 angiographically confirmed coronary atherosclerosis patients and 500 age and sex matched control individuals	−HCC was significantly higher in patients with angiographically confirmed coronary atherosclerosis compared with control individuals	^193^
Stress (bullying at school)	−HCC was assessed from scalp hair by ELISA	−The study included 659 11-year-old preadolescents	−Bully/victim status was linked to higher HCC, which in turn was associated with poorer executive function, highlighting the potential impact of chronic stress on preadolescent health and development	^194^

A meta-analysis by Li et al.^141^ of 13 studies across five countries (1455 participants) found a strong link between chronic stress and HCC and significant correlations emerged when stress was assessed via life events in the past 6 months, with HCC analyzed from 1-, 3- or 6-cm hair samples using LC−MS/MS. Collecting both psychological and biological stress data is vital for understanding the connections between stress, hypertension and mental well-being. Richards et al.^142^ explored these relationships in African American adults aged 65 years and older, finding that improved mental well-being correlated with reduced perceived stress and lower hypertension. In brief, HCC offers a reliable, noninvasive biomarker for chronic stress, eliminating the need for multiple daily biological fluid samples, making it a valuable tool in stress research and health assessment.

In real-world settings, hair cortisol and cortisone—markers of cumulative HPA axis activity—have been used to assess burnout and resilience in healthcare workers during the COVID-19 pandemic^143^. In Kenya, elevated hair glucocorticoids were linked to stress symptom variability in pregnant women, highlighting underlying socioeconomic and psychosocial stressors^144^. Real-world applications include refugee settings, post-conflict regions and occupational health, where conventional stress assessments are constrained by recall bias and cultural factors. Hair biomarkers enable early detection of at-risk individuals and evaluation of mental health interventions in underserved populations.

## The role of hair in forensic investigations

Owing to their ability to retain drugs, hair specimens are valuable in forensic science for sexual assault cases, drug offenses and child abductions. It also aids in species identification, homicides, burglaries, hit-and-run accidents, race determination and individual identification^145^. Unlike blood, hair reveals drug use over weeks or months, preserving evidence even after bodily fluids degrade.

### Molecular analysis for human identification

Circumstantial evidence such as fingerprints, DNA, hair and fibers links suspects to crime scenes, with hair playing a key forensic role. Shed hair without roots is analyzed using microscopy and mitochondrial DNA sequencing. Microscopy has a high false-positive rate, as stated in an FBI report^146^, while mitochondrial DNA provides statistical identity but is limited by maternal inheritance. Although DNA is the gold standard, it fails in cases involving identical twins. RNA analysis aids forensic pathology by revealing disease mechanisms, cause of death and serving as a diagnostic tool. It helps estimate wound age, postmortem interval and identify body fluids through cell-specific mRNA expression, complementing DNA analysis^147,148^. Previous reviews have covered the various applications of RNA in forensic science^149^.

Recent advancements in MS and bioinformatics have enabled researchers to use single amino acid polymorphism-containing peptides in hair proteomic datasets to infer nonsynonymous single-nucleotide polymorphisms alleles in genomes^145,150^. This profiling helps estimate the likelihood of certain alleles and assess biogeographic background. Parker et al.^145^ used MS-based shotgun proteomics to analyze hair shaft proteins from 66 European−American subjects, imputed 596 single-nucleotide polymorphisms alleles across 32 loci from 22 genes, and validated findings with Sanger sequencing, showing that hair proteins can serve as identity markers. Mason et al.^150^ utilized a 1-inch hair sample, suitable for forensic use, and applied an exome-driven approach for identifying genetically variable peptides (GVPs), whereas Parker et al.^145^ used a generic set of GVPs validated individually by Sanger sequencing. While exome-driven methods efficiently identify genetically valid GVP markers for initial surveys, Sanger sequencing remains crucial for confirming false positives.

Chu et al.^151^ studied single-inch hair samples from the head, arm and pubic regions, identifying GVP markers that remain consistent across body locations. According to the findings from these authors, these keratin markers do not vary by location. However, differences in keratin-associated and intracellular protein profiles were notable, aiding in distinguishing body locations. Adav et al.^11^, in their proof of concept research, used proteomics to analyze hair from males and females of different ethnicities (Chinese, Indian, Malay and Filipino, aged 20–80 years), finding distinct profiles in hair keratins and keratin-associated proteins. They highlighted hair the potential of proteomics in forensic science for distinguishing individuals by ethnicity, sex and age. However, they stressed the need for further validation, as protein extraction varied using different buffers. Optimizing protocols can improve data reliability, aiding forensic investigations and crime scene reconstruction. When combined with traditional microscopy, hair proteomics significantly enhances forensic analysis.

### Evidence in crime investigation

Drug-facilitated crimes (DFCs) use drugs to incapacitate victims for offenses such as robbery or sexual assault. Hair analysis is crucial in DFC and drug-facilitated sexual assault (DFSA) investigations, as it stores drug metabolites for months, offering a long-term record. Its noninvasive collection and stability make hair a valuable tool for forensic cases where timely evidence is difficult to gather.

#### DFSA cases

DFSA, a subcategory of DFCs, predominantly affects women, with studies in the Netherlands and New Zealand reporting 94% and 98% female victims, respectively^152,153^. In sexual assault investigations, biological materials such as semen, saliva, blood, vaginal fluid and hair serve as key evidence. Sexual assault investigations are multidisciplinary, involving sexual assault nurses, police, forensic scientists, healthcare specialists and the criminal justice community, where delays in sampling can degrade biological fluids but hair remains a stable specimen for drug exposure detection. The detection window for these fluids varies: vaginal fluid is detectable for 3−12 days and saliva for 3−7 days. Hair analysis has two key advantages: it retains drugs and metabolites long-term and it can detect multiple drugs even after a single dose. Drugs such as zolpidem, flunitrazepam and morphine have been detected in hair after single-dose intake^154^. The disposition of hypnotics such as triazolam, etizolam, flunitrazepam, nitrazepam and zolpidem after a single oral dose was assessed using LC–MS/MS^155^.

Immunoassays were once used for drug screening. Recently, researchers have employed sensitive MS to quantify drugs in hair samples. Common DFSA drugs include ethanol, benzodiazepines, barbiturates, opioids and gamma hydroxybutyrate (GHB), all of which can be detected in hair, even after low doses. Segmental hair analysis provides insights into drug use patterns, distinguishing between single-dose exposure and long-term use^156^. Techniques such as microsegmentation (0.4 mm segments) allow precise detection, such as determining the exact day of ingestion. Studies such as those by Wen et al.^157^ demonstrate the effectiveness of these methods in DFSA cases, detecting drugs long after exposure. Almofti et al.^154^ developed a eco-friendly extraction method to quantify 23 DFSA drugs, including scopolamine, which was detectable in hair 5 weeks post-exposure. These findings reinforce hair as a valuable biospecimen in drug-facilitated crime investigations.

#### Psychoactive substance abuse

Psychoactive substances alter brain function, affecting perception, mood, consciousness, cognition and behavior. In recent years, new psychoactive substances (NPSs) have surged, posing significant health risks. By 2022, over 930 NPSs were monitored in Europe, including synthetic cannabinoids, cathinones, opioids and benzodiazepines^158^. NPSs often evade drug laws. They are rapidly eliminated from bodily fluids, complicating toxicological confirmation. However, hair analysis offers a longer detection window for NPSs compared with urine. Studies using LC−MS have revealed the prevalence of NPSs in high-risk populations^159^. Using LC‒MS, Musshoff et al.^160^ developed a method for the simultaneous determination of 11 opioids and 4 metabolites (buprenorphine, codeine, fentanyl, hydromorphone, methadone, morphine, oxycodone, oxymorphone, piritramide, tilidine, tramadol and their metabolites bisnortilidine, nortilidine, norfentanyl and normorphine) in hair samples. Giorgette et al.^161^ identified 132 synthetic cannabinoids, 22 synthetic opioids and 28 substances among synthetic cathinones and stimulants. Barone et al.^162^ developed an LC‒MS method for the detection and quantification 127 NPSs in hair. Similarly, Zhai et al.^163^ used UPLC‒MS/MS to identify 75 phenethylamines and their derivatives in hair samples. Thus, LC−MS is a key technology for detecting NPSs and their metabolites in hair biospecimens, providing valuable information for forensic investigations.

#### Drugs of abuse

Drugs of abuse are substances misused for their psychoactive effects, potentially leading to addiction and health problems. While alcohol is legal, its misuse can cause serious consequences. Detecting drug metabolites in clinical and forensic settings provides reliable evidence of use. The Society of Hair Testing (SoHT) recommends methods such as metabolite analysis and metabolite-to-parent drug ratios for accurate detection. Alcohol biomarkers such as ethylglucuronide (EtG) and fatty acid ethyl esters in hair can signal chronic excessive consumption^164^. SoHT suggests a cutoff of 30 pg/mg of EtG in the 0–3 cm proximal segment of scalp hair to indicate chronic alcohol abuse. Fatty acid ethyl esters (ethyl myristate, palmitate, oleate and stearate) should be quantified together, with a cutoff of 0.5 ng/mg for their sum in the same hair segment to indicate chronic excessive alcohol consumption. In traffic-related crimes, hair analysis is useful for detecting alcohol abuse and assessing drug use history and therapy compliance through segmentation. Although not regulated globally, hair testing is permitted in certain states and used in Europe for criminal investigations and monitoring chronic drug use. Hair analysis, using techniques such as GC−MS and LC−MS, is increasingly used for drug detection owing to its sensitivity and specificity.

Hair analysis is popular in identifying drugs of abuse, such as 3,4-methylenedioxymethamphetamine (MDMA), cocaine and opiates, and is especially relevant in legal, criminal and workplace contexts. Seized MDMA tablets often contain various components, including 3,4-methylenedioxyamphetamine (MDA), amphetamine, ketamine, cocaine, caffeine, phencyclidine and ephedrine, indicating potential multidrug abuse^165^. Kronstrand et al.^166^ developed a rapid LC−MS/MS screening method for 14 abused drugs, including nicotine, morphine, amphetamine, MDMA, cocaine and diazepam, with detection limits of 3−70 pg/mg in hair. Gambelunghe et al.^167^ analyzed 90 hair samples from cocaine addicts, categorizing cocaine use as light (0.5–3 ng/mg), moderate (3.1–10 ng/mg) and heavy (10.1–40 ng/mg) based on cocaine, benzoylecgonine and related compounds. Pan et al.^168^ reviewed 9,083 cases including 1,992 traffic accidents, 6,787 drug abuses, 269 poisonings and 35 DFSAs, finding amphetamines were the most commonly abused drug, followed by opiates, ketamine and cocaine. Zhuo et al.^169^ analyzed 12 drugs of abuse in hair, revealing amphetamines as the most frequently abused (142 cases), followed by opiates, ketamine and cocaine. Chas et al.^170^ found cocaine was the most commonly used drug among 69 volunteers, followed by MDMA. Mannocchi et al.^171^ used UHPLC−MS/MS to detect 87 NPS and 32 classic illicit drugs in hair and nails. Musshoff et al.^172^ analyzed over 100 drugs, including narcotics and antidepressants, in untreated Caucasian hair, providing a valuable reference database. Hair segmentation analysis is also valuable in workplace drug testing, license reinstatement, drug abuse history, drug-related death investigations, drug-facilitated crime and therapy compliance^173^. Substance abuse during pregnancy is a concern, with potential long-term effects on children. Pregnant women often use illegal drugs, with opioids and marijuana use on the rise^174,175^. Prenatal drug exposure can lead to developmental issues in children.

The strength of hair testing in forensic science lies in its ability to reconstruct long-term exposure histories, supporting investigations into abuse, neglect, substance use and postmortem toxicology. Its durability and ease of collection make it particularly useful in legal cases and situations where conventional samples are unavailable. Advancements in techniques such as LC‒MS/MS, ICP‒MS, proteomics and stable isotope analysis have expanded the forensic utility of hair, enabling detection of drugs, metals, isotopes and DNA from small quantities of samples. Isotopic signatures can indicate dietary patterns and geographic origin, supporting investigations involving unidentified remains, human trafficking and historical migration. Despite its effectiveness, hair testing is not yet accepted by the International Olympic Committee or the World Anti-Doping Agency for doping control but is used to verify self-reported drug histories. Hair color and melanin levels affect drug testing, requiring proper normalization and statistical adjustments.

### Environmental exposure monitoring

Hair analysis provides a historical record of exposure to exogenous compounds such as drugs, alcohol, pesticides and toxic metals, even after they disappear from blood, urine or oral fluids. Exposure to multiple chemicals is linked to health risks such as AD, asthma and cancer^176,177^. While blood and urine are commonly used in exposomics, their chemical composition is influenced by daily activities and circadian rhythms. In contrast, hair accumulates chemical compounds over time, making it ideal for long-term exposure monitoring. Studies have identified metabolites of chemicals such as di(2-propylheptyl)phthalate in hair^178^, and HRMS-based screening has detected 167 chemical compounds in hair, demonstrating its potential in environmental exposure studies^179^.

#### Ethical considerations in the use of hair biospecimens

The use of hair biospecimens in clinical and research settings presents unique ethical challenges, particularly when involving vulnerable populations such as children, pregnant individuals, incarcerated persons or those with cognitive impairments^180,181^. These individuals may have limited capacity to provide fully informed or voluntary consent, necessitating enhanced safeguards, including adapted consent processes, legal guardianship involvement and independent ethical oversight. Analyzing pubic hair in forensic or clinical contexts raises specific ethical concerns related to privacy, consent and dignity. Owing to the intimate nature of the sampling site, explicit, informed consent is essential, including a clear explanation of the purpose, procedures and alternatives. Hair analysis can reveal sensitive longitudinal data, such as substance use or environmental toxin exposure, that may have unintended legal, social or psychological consequences. In such cases, disclosure of incidental findings must be carefully managed to avoid harm, stigma or discrimination. Privacy protections must be rigorously enforced and data use should be limited to clearly defined purposes. The potential for genetic and metabolic data to be repurposed outside clinical contexts heightens privacy risks^182^. Cultural sensitivity is also critical as hair may hold symbolic importance in certain communities. Analytical validity must be maintained through strict protocols, contamination control and careful interpretation that accounts for biological variability and potential external contamination. Thus, key ethical safeguards include context-sensitive informed consent, strict data protection and access controls, policies preventing discriminatory or stigmatizing use of genetic and toxicological data, adherence to legal and regulatory standards and respect for cultural and individual autonomy in biospecimen use^183–188^. In precision medicine and forensic contexts, upholding these principles is essential to protecting the rights, dignity and autonomy of vulnerable individuals throughout the research and application process.

Beyond ethical guidelines, SoHT emphasizes standardized protocols to ensure accuracy and reliability in hair analysis. Key recommendations include collecting proximal scalp hair, preserving strand alignment, storing samples dry at room temperature and documenting hair characteristics and treatments. Segmental analysis enables retrospective exposure assessment, while body hair is discouraged due to inconsistent growth. Validated decontamination procedures are essential to reduce contamination and preserve analyte integrity. Quantification should rely on chromatographic techniques with MS for specificity, with immunoassays limited to preliminary screening and requiring confirmation. Quality assurance must include internal standards and external proficiency testing. Interpretation should account for biological variability, cosmetic history, environmental exposures and the specific forensic or clinical context.

According to the SoHT, ethanol cannot be directly measured in hair; instead, its metabolites EtG and ethyl palmitate (EtPa) are used as biomarkers of alcohol consumption. EtG is optimally extracted from powdered hair using aqueous methods, while EtPa requires prewashing with a nonpolar solvent. Quantification should be performed using validated LC−MS/MS methods. Abstinence is indicated by EtG ≤5 pg/mg and EtPa ≤120–150 pg/mg; concentrations above these suggest repeated alcohol use. Chronic excessive consumption is defined by EtG ≥30 pg/mg and EtPa ≥350–450 pg/mg. Similarly, SoHT provides guidelines for drugs of abuse testing in hair.

## Challenges and limitations

Over the past decade, hair analysis has become valuable in diagnostics, therapeutics and forensic toxicology. While urine and blood are reliable for acute exposures, hair captures long-term substance exposure, offering insights over extended periods. However, hair analysis presents several methodological and interpretive challenges that limit its consistency and reliability across clinical and forensic settings. Hair shafts are susceptible to external contamination from environmental pollutants, drugs, smoke and personal care products, which can adsorb to or infiltrate the hair shaft, confounding the distinction between endogenous and exogenous substances. Decontamination procedures, such as sequential solvent washes, are necessary but not standardized and may either fail to fully remove contaminants or strip endogenous compounds. Interindividual variability in hair growth rate, pigmentation and cosmetic treatments further complicates analysis and interpretation. Sampling inconsistencies—including anatomical collection site, hair length, orientation and storage—add to the variability in results across studies and laboratories. Additionally, ultraviolet exposure can degrade melanin, affecting analyte binding and diffusion. In forensic cases, the lack of hair roots precludes nuclear DNA analysis, requiring reliance on less discriminatory mitochondrial DNA or protein-based approaches, which also suffer from a lack of standardization.

Despite its value as a noninvasive and longitudinal matrix, hair analysis has several inherent limitations that restrict its clinical and diagnostic applicability. It is less effective for detecting recent or sporadic exposures due to a 1–2 week incorporation lag. Hair lacks temporal precision, making it more suitable for retrospective exposure profiling rather than real-time monitoring. Biomarker concentrations in hair are typically lower than in other biological matrices, necessitating the use of highly sensitive and often costly analytical techniques such as LC−MS/MS, which may not be accessible in all settings. Additionally, hair biospecimens yield low and variable amounts of DNA or RNA, limiting their utility in genomics and transcriptomics. In neurodegenerative diseases and other systemic conditions, hair may primarily reflect peripheral changes, reducing its diagnostic specificity. The absence of widely accepted interpretive thresholds, clinical validation and regulatory standards further limits its integration into routine medical or public health practice.

## Knowledge gaps and future perspectives

Despite growing applications of hair biospecimens in precision medicine and forensic science, several critical knowledge gaps persist. Biologically, the mechanisms governing the incorporation of drugs, toxins and biomarkers into hair remain incompletely understood, particularly across diverse hair types, ethnicities and physiological conditions such as pregnancy or disease. Distinguishing true internal exposure from external contamination remains a key challenge. Additionally, variability in hair growth rates and metabolite deposition complicates temporal resolution and limits accurate reconstruction of exposure timelines.

Analytically, the field lacks standardized protocols for collection, processing and interpretation, leading to inconsistencies across laboratories. The sensitivity and specificity of current methods vary, especially at low analyte concentrations or in pediatric populations. Clinically, many biomarkers detected in hair remain unvalidated, and their correlations with disease states or therapeutic outcomes are poorly established. Ethically, frameworks for informed consent, data governance and return of incidental findings, especially for vulnerable populations, are underdeveloped. Cultural sensitivities and the risk of misuse (for example, discrimination or stigmatization) further underscore the need for rigorous ethical oversight. Addressing these gaps is essential for responsible and effective integration of hair biospecimens into clinical and forensic practice.

Variability in hair characteristics such as pigmentation, cosmetic treatments, environmental factors and growth rate, poses a persistent challenge in hair analysis, influencing analyte incorporation, stability and temporal interpretation. Melanin content, the lipophilicity, basicity and polarity of a drug and its metabolites have been shown to affect drug binding, introducing potential bias related to hair color. Cosmetic and chemical treatments (for example, bleaching, dyeing or perming) and environmental factors (for example, UV light) can alter the structure of the hair shaft, compromising analyte integrity, stability and extraction efficiency. Additionally, growth rate variability by factors such as age, health status and anatomical site complicates the reconstruction of exposure timelines. In forensics, diagnostics, clinical sciences and research contexts, these variabilities are often underrecognized. Existing analytical practices, including pre-analysis screening, segmental analysis and normalization may provide limited mitigation. To minimize the impact of these sources of variability, fundamental research is needed to better understand their effects and to develop robust reference databases that account for these factors in both forensic and diagnostic contexts. Integrating this knowledge into standardized analytical protocols is essential to improve the validity, reliability, and equity of hair biospecimen analysis. Recent advancements in MS, microscopy and spectroscopy techniques have greatly improved our understanding of hair biology. Technologies such as multiphoton fluorescence, synchrotron radiation X-ray and FTIR enable noninvasive disease diagnosis, monitoring and treatment customization. Enhanced MS sensitivity and molecular bioinformatics are advancing omics-based approaches, identifying disease biomarkers, drug exposure and nutritional status. These technologies improve detection limits and distinguish between acute and chronic exposure, with hair reflecting long-term hormonal changes such as cortisol, aiding mental health assessments.

Hair analysis is widely used in biomonitoring to study nutrition, toxin exposure and drug abuse. However, interpreting elemental analysis, especially for metals such as Se, Pb, Cu and Zn, remains complex as they influence disease processes and conditions such as cancer and atherosclerosis. ICP−MS enhances the accuracy of trace element detection. Segmental hair analysis offers insights into health changes, treatment efficacy and exposure over time, making it valuable for personalized medicine, pharmacokinetics and forensic toxicology. The integration of MS and bioinformatics is advancing precision medicine by enabling personalized, noninvasive diagnostics. Bioinformatics helps interpret MS data, providing a comprehensive view of health, while machine learning and artificial intelligence improve disease detection and personalized treatment plans.

Distinguishing endogenous from exogenous analytes remains a major challenge, requiring systematic efforts such as isotopic labeling; mechanistic studies of incorporation via sweat, sebum and blood; and the development of reference databases. Standardized thresholds for interpreting toxicological findings are also lacking. The effects of cosmetic treatments on a broad range of analytes are underexplored and warrant systematic investigation. Assumptions of uniform hair growth and analyte deposition are often inaccurate; real-time in vivo labeling and algorithms to account for growth variability and shedding are needed. Integration of hair-based metabolomics, proteomics and epigenomics with genomics and exposomics remains limited. Furthermore, longitudinal, noninvasive monitoring of stress, hormones and disease biomarkers, linked with digital phenotyping tools such as wearables, offers a promising but largely untapped opportunity for individualized risk profiling.

Several underexplored and controversial areas in hair analysis warrant further investigation. These include the mechanisms of drug incorporation and the challenge of distinguishing endogenous uptake from environmental contamination. Epigenetic markers and hair-based metabolomics/proteomics offer promising avenues for noninvasive diagnostics but require further validation. Concerns over bias related to hair type and ethnicity, as well as the potential for detecting environmental pollutants such as microplastics, highlight the need for standardized, inclusive methodologies.

## Supplementary information

Supplementary Table 1
