# Supplementary material for: The multifaceted role of hair as a biospecimen: recent advances in precision medicine and forensic science
Source: Exp Mol Med. 2025 Oct 27;57(10):2234–50. doi: 10.1038/s12276-025-01548-4 (PMC12586478; doi:10.1038/s12276-025-01548-4)
Supplement: Supplementary file 1 — Supplementary Table 1 [file 12276_2025_1548_MOESM1_ESM.docx]

**The Multifaceted Role of Hair as a Biospecimen: Recent Advances in Precision Medicine and Forensic Science**

Sunil S Adav^a*^ and Kee Woei Ng^a,b*^

^a^School of Materials Science and Engineering, Nanyang Technological University, Singapore, Singapore

^b^Nanyang Environment and Water Research Institute, Nanyang Technological University, Singapore, Singapore

Table S1. Application of FTIR in Medicine Using Hair as a Biosample: Wavenumber Assignments for Biomolecules and Intermolecular Bonds in Cancer and Hair Analysis.

| Wavenumber | Definition | Biochemical molecule | References |
| --- | --- | --- | --- |
| 3294–3328–3330 | Amide A: Predominantly linked to the N–H stretching vibrations of proteins, with its characteristics influenced by intermolecular hydrogen bonding. It also includes contributions from the O–H stretching vibrations of polysaccharides. | Mostly proteins | ^183^ |
| 3129 | Amide B: Arises from the N–H stretching vibrations of proteins. | Proteins | ^184^ |
| 3060–3065 | Amide B: Corresponds to the C–N and N–H stretching vibrations of proteins. | Proteins | ^184^ |
| 3011–3015 | Olefinic, Refers to the C–H stretching vibrations of HC=CH groups. | Unsaturated lipids | ^184^ |
| 2957–2962 | Primarily associated with CH₃ antisymmetric stretching. | Mainly lipids | ^183^ ^185^ |
| 2920–2929 | Primarily related to CH₂ antisymmetric stretching. | Mainly lipids | ^183,184,186^ |
| 2907 | CH₂ and CH₃ stretching vibrations associated with phospholipids, cholesterol, and creatine. | Mainly lipids | ^186^ |
| 2871–2875,2873 | CH_3_ antisymmetric stretching | Protein side chains and lipids, with additional contributions from carbohydrates and nucleic acids. | ^183,184,186^ |
| 2850–2858 | CH_2_ symmetric stretching | Protein side chains and lipids, with additional contributions from carbohydrates and nucleic acids. | ^183,184,186^ |
| 2843 | C–H stretching | Mianly lipids | ^186,187^ |
| 1720–1732  1740–1745 | C = O stretching Saturated ester | Cholesterol esters, phospholipids, fatty acid ester functional groups in lipids | ^186^ |
| 1710−1716 | Antisymmetric stretching C = O | RNA and purine base | ^184^ |
| 1705−1690 | C = O antisymmetric stretching vibrations: | RNA, DNA | ^184^ |
| 1685 | β‐turn protein secondary structure | Protein | ^188^ |
| 1653, 1654 | Amide I: Comprises approximately 80% C=O stretching, 10% N–H bending, and 10% C–N stretching vibrations in proteins. | Protein α‐helix | ^186^ |
| 1648 | Amide I (C = O) (C–N) (N–R2) | Protein | ^187^ |
| 1630−1640, 1684–1656 | Amide I: C = O (80%) and C–N (10%) stretching, N–H (10%) bending vibrations: | Proteins β‐structure | ^183,184,186^ |
| 1610, 1578 | C4‐C5 and C = N stretching in imidazole ring of DNA, RNA | DNA | ^184^ |
| 1554 | Overall protein absorbance | Protein | ^183,185^ |
| 1540–1550  1452, 1388 | Amide II: N–H (60%) bending and C–N (40%) stretching vibrations | Protein α‐helix | ^184^ |
| 1530–1535 | Amide II: N–H (60%) bending and C–N (40%) stretching vibrations: | Protein β‐structure | ^184,187^ |
| 1452, 1453,1455–1467 | CH_2_ bending vibrations: | lipids and proteins | ^183^ |
| 1370−1400–1452, 1455 | CH_2_ and CH_3_ deformation vibrations due to lipid contribution | lipids, proteins | ^184^ |
| 1396 | COO– symmetric stretching vibrations of amino acid side chains | fatty acids | ^185,186^ |
| 1376–1378 | Methyl or CH_3_, CH_2_ wagging | lipids/proteins | ^187^ |
| 1330−1200  1284, 1280 | Amide III | Proteins | ^184^ |
| 1310  1304 | Peptide side‐chain vibrations  Deformation N‐H cytosine |  | ^183,187^ |
| 1230−1244 | PO_2_‐ antisymmetric stretching's | Completely hydrogen bonded: mainly nucleic acids with little contribution from phospholipids | ^183,184,186^ |
| 1147, 1152–1156  1124 | CO–O–C antisymmetric stretching vibrations characteristic of glycogen and nucleic acid ribose, along with νC–O stretching modes of carbohydrates. |  | ^183,186,187^ |
| 1090–1084 | PO_2_‐ symmetric stretching vibrations | : RNA, DNA | ^184,186^ |
| 1060, 1050 | C–O stretching vibrations | deoxyribose/ribose DNA, RNA | ^187^ |
| 1045–1050 | CO stretching vibrations associated with carbohydrates and glycogen, as well as deoxyribose and ribose in nucleic acids. |  | ^183^ |
| 1021–1041 | C–O stretching combined with C–O bending of the C–OH groups in carbohydrates, oligosaccharides, and polysaccharides. | Mainly from glycogen | ^183,186,188^ |
| 996, 995, 994 | C–O stretching vibrations from the RNA ribose chain and other carbohydrates. |  | ^184,188^ |
| 975.1, 976−875 | C–N^+^–C stretching vibrations in nucleic acids (DNA, RNA), along with ribose phosphate backbone vibrations in RNA and phosphate monoesters. | DNA, RNA | ^186,188^ |
| 958.7–954.9, 962.6 | νCC of the DNA backbone | DNA | ^188^ |
| 950‐ | C–C vibrations from nucleic acids | DNA | ^185^ |
| 957 | CH_3_ deformation (lipid, protein) | Lipid, protein | ^184^ |
| 936 | C–C residue *α*‐helix |  |  |
| 916.3, 925–929 | Sugar vibrations in the backbone of DNA‐Z form | Ribose ring | ^183,188^ |
| 921 | C–C stretch proline |  |  |
| 895.1–899.9 | Deoxyribose ring |  | ^188^ |
| 898 | C–C stretch residue |  |  |
| 889.3 | DNA band or C‐C, C‐O deoxyribose | Fatty acid, saccharide | ^187,188^ |
| 870 | C‐DNA |  |  |
| 855 | Vibrations in N‐type sugars in nucleic acid backbone |  | ^183^ |
| 853 | Ring breathing Tyr‐C–C stretch proline |  | ^184^ |
| 767–786 | C–C and C–N stretch PO_3_ ^2−^ stretching |  | ^184^ |

References:

183 Aksoy, C. & Severcan, F. Infrared spectroscopy and imaging in stem cells and aging research. *Methods Mol Biol* **2045**, 201-215, doi:10.1007/7651_2018_119 (2019).

184 Aksoy, C. & Severcan, F. Role of vibrational spectroscopy in stem cell research. *J. Spectrosc.* **27**, 167-184 (2012).

185 Cao, J. *et al.* The characterisation of pluripotent and multipotent stem cells using Fourier transform infrared microspectroscopy. *Int. J. Mol. Sci.* **14**, 17453-17476 (2013).

186 Igci, N. *et al.* Application of Fourier transform infrared spectroscopy to biomolecular profiling of cultured fibroblast cells from Gaucher disease patients: A preliminary investigation. *Adv Clin Exp Med* **26**, 1053-1061, doi:10.17219/acem/65784 (2017).

187 Barnas, E. *et al.* Simultaneous FTIR and Raman spectroscopy in endometrial atypical hyperplasia and cancer. *Int. J. Mol. Sci.* **21**, 4828 (2020).

188 Ami, D. *et al.* Embryonic stem cell differentiation studied by FT-IR spectroscopy. *Biochim Biophys Acta* **1783**, 98-106, doi:10.1016/j.bbamcr.2007.08.003 (2008).
